# Dynamicity in Host Metabolic Adaptation Is Influenced by the Synergistic Effect of Eugenol Oleate and Amphotericin B During *Leishmania donovani* Infection *In Vitro*

**DOI:** 10.3389/fcimb.2021.709316

**Published:** 2021-08-03

**Authors:** Amrita Kar, Adithyan Jayaraman, Avanthika Kumar, Santanu Kar Mahapatra

**Affiliations:** ^1^Department of Biotechnology, School of Chemical and Biotechnology, Shanmugha Arts, Science, Technology & Research Academy (SASTRA) Deemed to be University, Thanjavur, India; ^2^Department of Paramedical and Allied Health Sciences, Midnapore City College, Midnapore, India

**Keywords:** eugenol oleate, amphotericin B, nitric oxide, immune metabolism, p38MAPK

## Abstract

Immune metabolic adaptation in macrophages by intracellular parasites is recognized to play a crucial role during *Leishmania* infection. However, there is little accessible information about changes in a metabolic switch in *L. donovani* infected macrophages. In previous studies, we have reported on the anti-leishmanial synergic effect of eugenol oleate with amphotericin B. In the present study, we demonstrated that glycolytic enzymes were highly expressed in infected macrophages during combinatorial treatment of eugenol oleate (2.5 µM) and amphotericin B (0.3125 µM). Additionally, we found that the biphasic role in arachidonic acid metabolite, PGE2, and LTB4, is released during this treatment. *In vitro* data showed that COX-2 mediated PGE2 synthesis increased significantly (p<0.01) in infected macrophages. Not only was the level of prostaglandin synthesis decreased 4.38 fold in infected macrophages after treatment with eugenol oleate with amphotericin B. The mRNA expression of PTGES, MPGES, and PTGER4 were also moderately expressed in infected macrophages, and found to be decreased in combinatorial treatment. In addition, NOS2 expression was activated by the phosphorylation of p38MAPK when combination-treated macrophages were promoted to kill intracellular parasites. The findings of the present study indicate that the synergism between eugenol oleate and amphotericin B could play an important role in immune metabolism adaptation with a concomitant increase in host immune response against the intracellular pathogen, *L. donovani*.

## Introduction

Visceral leishmaniasis (VL) is caused by the intramacrophagic protozoan parasite of *Leishmania donovani* and *L. infantum*. Initialization of infection is started inside the host body during insertion of flagellated promastigote forms in a sandfly blood meal. After inoculation, promastigotes are phagocytosed by macrophages and differentiated into intracellular amastigotes. This reciprocity between parasite proliferation and host immunological responses is pivotal for infection establishment and disease amelioration (Gupta et al., 2021). As per WHO information, 0.7 to 1 million new VL cases are reported annually.

The innate immune response of our body recruits different types of cellular subsets to fight against pathogens. These cellular subsets function in a complex manner to evade various invading factors and generate cellular homeostasis to protect the host body. Cellular subsets include circulating lymphocytes (T-cell, B-cell, and NK cells) and monocytes that can establish either into dendritic cells or macrophages. The plasticity of macrophages can develop phenotypical changes by a process called ‘polarization’ (Mantovani et al., 2004; Sica and Mantovani, 2012). Macrophage polarization can switch in response to the endogenous stimuli during infection and signals both pathogenic and protective functions (Mills, 2012; Boche et al., 2013). Thereby, it was mainly classified into two subtypes like classically activated protective M1 polarized macrophages and alternatively activated pathogenic M2 macrophages (Stein, 1992; Doyle et al., 1994). The procedure of macrophage polarization is regulated by metabolic reprogramming with bioenergetics demands to keep obligatory immunological functions. This cellular metabolic signaling relies on the consumption of carbohydrates, fatty acids, proteins, or amino acids (O’Neill and Pearce, 2016). It has been reported that macrophages infected *via L. donovani* and *L. amazonensis* parasite induced M2 polarized profile, which upregulated oxidative phosphorylation (Zhang et al., 2010; Pearce and Pearce, 2013; Osorio et al., 2014). Likewise, *L infantum* also induced transcriptomic gene expression of glycolytic enzymes during the early phase of infection but not in the late phase of infection (Moreira et al., 2015). Additionally, glycyrrhizic acid suppressed COX-2 mediated prostaglandin secretion in *L. donovani* infected macrophages (Bhattacharjee et al., 2012). Therefore, the adaptation in metabolic shifting during leishmaniasis could implicate an important target to elicit host immune responses.

Several chemotherapeutic regimens were developed due to a lack of proper vaccine development and vector control program. This emphasizes the urgent need to develop new, safe, cheap, low dosage, and short duration drug alternatives. Combination therapy could be a better option to treat visceral leishmaniasis. Considering the limitations of cost, effectiveness, and drug resistance in chemotherapeutics, alternative approaches with newly synthesized compounds are required. Likewise, eugenol, a plant-derived essential oil has exhibited anti-bacterial, anti-viral, anti-cancer, and anti-leishmanial activity. Eugenol oleate, a eugenol derivative, was reported as an immunomodulatory molecule with less toxicity (Charan Raja et al., 2021). Moreover, it was reported that eugenol oleate has shown synergistic anti-leishmanial property in combination with amphotericin B (x∑FIC = 0.456) against experimental visceral leishmaniasis (Kar et al., 2021).

The hijacking of the metabolic signaling pathways is correlated with an apparent contour that generates parasite survivability and elimination. The present study discusses the bidirectional alteration in immunometabolic profiles after the treatment of eugenol oleate in combination with amphotericin B in *L. donovani* infected macrophages.

## Materials and Methods

### Chemicals

Standard antileishmanial drugs, chemicals for eugenol oleate synthesis, and others were purchased from Sigma, Alfa Aesar, and Merck Chemicals. Cell and parasite culture media (RPMI-1640, M199) were purchased from HiMedia. FBS and antibiotics were purchased from Gibco; ELISA kits for PGE2 and LTB4 were obtained from R & D systems. Griess reagent and sequence-specific oligos were purchased from IDT. cDNA synthesis kit, RT-PCR chemicals, real-time PCR chemicals were procured from Fermentas and TAKARA Bio.

### Animals and Parasites

*Leishmania donovani* strain (MHOM/IN/1983/AG-83) was cultured in M199 medium with 10% FBS and 1X Pen-Strep at 22°C. Thio-glycolate broth injected BALB/c mice-derived macrophages were extracted and cultured in RPMI medium with 10% FBS at 37°C in a 5% CO_2_ incubator (Cell Xpert, Eppendorf). All animals used for the macrophage isolation received prior approval from the Institutional Animal Ethical Committee, Shanmugha Arts, Science, Technology and Research Academy (SASTRA) deemed university (Approval no: 489/SASTRA/IAEC/RPP, dated 9/9/2017; 612/SASTRA/IAEC/RPP, dated 10/08/2019).

### Preparation of Eugenol Oleate

Eugenol oleate was synthesized from eugenol and oleic acid as described earlier (Kar et al., 2021). In brief, oleic acid (1.8 mmol) was dissolved in 5 ml of dry DMF at 0°C in stirred conditions. After that, EDCI.HCl (1.65 mmol) and DMAP (0.15 mmol) were added and stirred for 30 min at 0°C. Hereafter, eugenol (1.5 mmol) was added and stirred. After completion of the reaction, 10 ml distilled water was added to the reaction mixture and stirred for 30 min. The organic portion was then extracted by portioning with EtOAc (3 X 10 ml) and dried over anhydrous Na_2_SO_4_. The organic mass was thus obtained by concentrating under reduced pressure and was purified by column chromatography (Charan Raja et al., 2021; Kar et al., 2021).

### Estimation of PGE2 and LTB4 Release by Sandwich ELISA

Isolated peritoneal macrophages infected with *L. donovani* parasite and treated with eugenol oleate and amphotericin B in combination as well as monotherapy also. In this study, we used a concentration of eugenol oleate at 2.5 µM in combination with 0.3125 µM of amphotericin B (AmpB) as described in our earlier report (Kar et al., 2021). After 24 hr supernatant was collected and the release of PGE2 or LTB4 was analyzed by a sandwich ELISA kit (R & D systems) as per the manufacturer’s instructions (Bhattacharjee et al., 2012).

### Isolation of mRNA and Semi-Quantitative and Quantitative qPCR

Total mRNA was extracted from macrophages by using Trizol (Invitrogen) and 1 µg of mRNA was used as a template for cDNA synthesis. GAPDH was used as a reference. Sequences of primer are listed in **Supplementary Table S1**. The amplification conditions were as follows: 35 cycles for 5 min at 95°C, 30 sec at 95°C, 30 sec at 62°C, 1 min at 72°C, and 10 min at 72°C. Amplified PCR products were then run on 1.2% agarose gel. Real-time PCR was performed by using SYBR green mix and obtained CT values. Relative quantification of studied genes was normalized with housekeeping gene (GAPDH) and expressed as mRNA fold change with uninfected control group by 2^-ΔΔCT^ method (Kar et al., 2021).

### Immunoblot Analysis

Cell lysates from different groups were prepared as described previously (Charan Raja et al., 2021). Afterward, 50 µg of protein was loaded in each lane in 10% SDS PAGE and transferred onto the PVDF membrane. Thereafter, that membrane was blocked by 5% bovine serum albumin in Tris-buffered saline (TBS) and immunoblotting analysis was carried out as described previously.

### *In Vitro* Anti-Amastigote Activity in Presence of Inhibitors

To investigate the anti-amastigote activity of eugenol oleate and its combinatorial effect with amphotericin B in the presence of inhibitors against intracellular *L. donovani* parasite, BALB/c derived peritoneal macrophages (1 × 10^5^) were plated in 200 μl of culture media per well in 8 well chamber glass slide and allowed for cell differentiation at 37°C in 5% CO_2_ containing incubator. Macrophages were infected with the stationary phase of AG83 promastigotes at 1:10 (macrophage: parasite) ratio for 4 h and the infected cells were kept another 20 h for amastigotes maturation and multiplication within macrophages. Macrophages were pretreated with a COX-2 inhibitor (NS-398), p38 MAPK inhibitor (SB203580), and NO inhibitor (LNMMA) for 1 h followed by the treatment in combination with Eugenol oleate (2.5 μM) and amphotericin B (0.3125 μM) were added and incubated mixture for 48 hr. After removing the culture medium, cells were washed twice with PBS and fixed with methanol. Then the chambered slides were stained with Giemsa. The intracellular amastigotes were measured per 100 macrophages by using an Olympus (BX43) microscope at 1000X magnification and resolution in oil immersion for each group (Charan Raja et al., 2021; Kar et al., 2021).

### Statistical Analysis

All the *in vitro* experiments were performed in triplicate using BALB/c derived peritoneal macrophages. The data were presented as mean ± SD. Two-way ANOVA followed by Tukey’s multiple comparison test were used to check the significant difference among all the groups using GraphPad Prism 6.

## Results

### Assessment of the Expression of Glycolytic Enzymes

**Figure 1:**
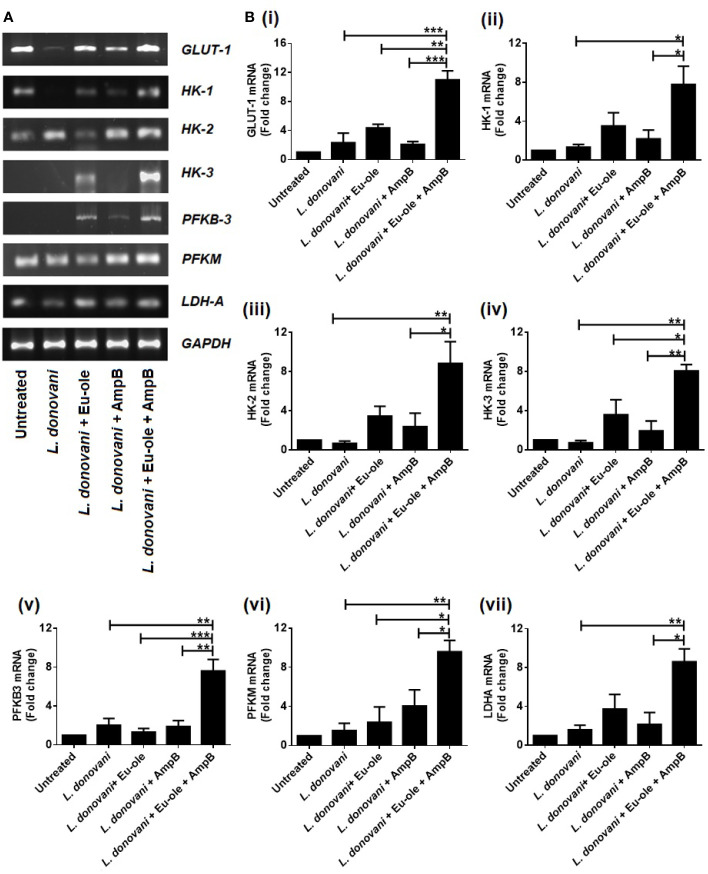
Alteration in glycolytic enzymes profile during combinatorial treatment. Peritoneal macrophages infected with *L. donovani* (1:10 ratio). After 24 hr of post infection, treatment was given to infected macrophages and after 6 hr, cells were collected in Trizol to determine the mRNA expression of glycolytic enzymes by RT PCR **(A)**. and the transcription levels of glycolytic genes *GLUT-1, HK1, HK-2, HK-3, PFKM, PFKB3* and *LDHA* were analysed by quantitative Real Time PCR (**B** i-vii). The results were expressed as mean ± SD. *p *<* 0.05, **p *<* 0.01, ***p *<* 0.001, significant differences between the indicated groups.

Immunemetabolism is correlated with the metabolic fluxes inside the cell. When the *Leishmania* parasites infect cells, cellular metabolic states are associated with the alteration in the cytokine-mediated functional types of macrophages. M1 macrophages mainly depend on the glycolysis bioenergetics pathway where glucose converts into pyruvate, either reduced in lactate or entered into the TCA cycle by several intermediate enzymes (Semba et al., 2016). In the present study, we investigated the expression of glycolytic enzymes by RT PCR data. We evaluated the expression of glucose transporter 1 (GLUT-1), isoforms of hexokinase (HK1, HK2, and HK3), phosphofructokinase 2 (PFKM), 6-phosphofructo-2-kinase/fructose- 2,6-biphosphatase 3 (PFKB3), and lactate dehydrogenase A (LDHA) transcripts. The expression was analyzed in BALB/c derived peritoneal macrophages challenged with stationary phase *L. donovani* parasites to establish the mechanisms accountable for energy metabolic profile recognition. After 24 hr of infection, cells were treated with 2.5 µM of eugenol oleate in combination with 0.3125 µM of amphotericin B *in vitro.* The levels of GLUT-1, HK-1, HK-2, HK-3, PFKM, PFKB-3, and LDHA were studied by using the semi-quantitative RT-PCR method. We found that the mRNA expression of glycolytic enzymes was upregulated in combination-treated macrophages than in monotherapy-treated cells (**Figure 1A**).

Similarly, these results were reconfirmed by using the real-time quantitative PCR method. The level of GLUT-1, one of glucose transporter was more significantly highly expressed 4.74 fold in combination than the infection set. While the expressions of the isoforms of hexokinase were significantly upregulated at 5.84, 13.03, and 11.33 fold in combination-treated macrophages for HK-1, HK-2, and HK3 respectively [**Figure 1B** (i-iv)]. The expression of PFKM and PFKB3 were significantly increased at 6.23 and 3.76 fold in combination therapy than infected macrophages [**Figure 1B** (v-vi)]. Interestingly, the expression of LDHA was increased 2.34 and 1.34 fold in eugenol oleate and amphotericin B treated cells compared to the infected macrophages. While in combination therapy, there was a significant induction of 5.39 fold of LDHA transcript compared to the infected set [**Figure 1B** (vii)]. Collectively, it could be suggested that *L. donovani* infection and monotherapy treated macrophages failed to upregulate the mRNA expression of glycolytic enzymes compared to the combination therapy of eugenol oleate and amphotericin B [**Figure 1B** (i-vii)]. Recently, we reported that our combination of eugenol oleate and amphotericin B was able to increase T-cell proliferation and proinflammatory cytokines against experimental visceral leishmaniasis *in vivo* (Kar et al., 2021). However, it has also been reported that murine immune cells upregulated glucose uptake and glycolysis by MAPK signaling (Marko et al., 2010). The induction of proinflammatory cytokines might upregulate the glycolysis pathway and a similar trend was observed in the present study after the use of combination therapy in *L. donovani* infected macrophages.

### Variation of the Expression in Lipid Metabolites During Treatment Due to Infection Acquisition

**Figure 2:**
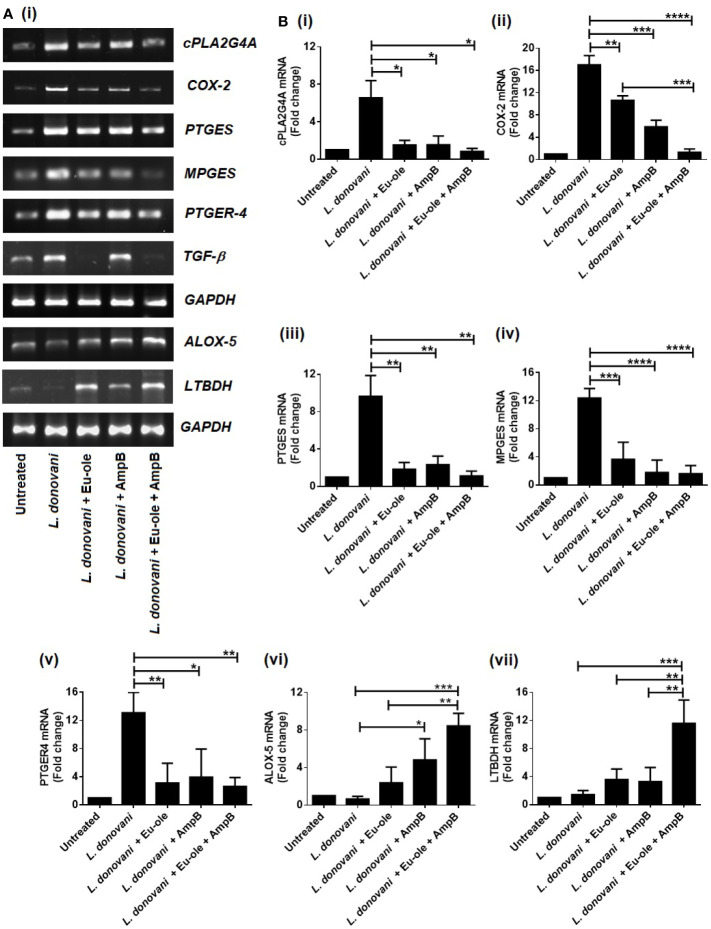
Biphasic expression in arachidonic acid enzymes profile during combinatorial treatment. Macrophages were infected with *L. donovani* (1:10 ratio). After 24 hr of post infection treatment was given in monotherapy as well as in combination therapy to infected macrophages. After 6 hr, cells were collected in Trizol to determine the mRNA expression of arachidonic acid enzymes were analysed by RT PCR **(A)** and the transcription levels of AA genes *cPLA2g4A, COX-2, PTGES, MPGES, PTGER4, TGF-β*, and *ALOX-5, LTBDH* were analysed by quantitative real time PCR (**B** i-vii). The results were expressed as mean ± SD. *p *<* 0.05, **p *<* 0.01, ***p *<* 0.001, significant differences between the indicated groups.

After the ingestion of obligatory parasites into the macrophages, the affected host body shows alteration in macrophage phenotypical changes through intracellular parasitism. Lipid mediators in arachidonic acid (AA), comprising leukotrienes and prostaglandin emerged in a signaling pathway during *Leishmania* infection (Bozza et al., 2009). Therefore, we checked the expression of arachidonic acid metabolism enzymes by murine exudate peritoneal macrophages infected with *L. donovani* parasites. The mRNA expression of cytosolic phospholipase A2 group IVA (cPLA2g4A) mediated prostaglandin E2 synthase (PTGER4, PTGES) and microsomal prostaglandin synthase (MPGES) were more enhanced in infected macrophages than in combinatorial treatment. On the other hand, the level of mRNA expression in arachidonate 5-Lipoxygenase (ALOX-5) and leukotriene B4 dehydrogenase (LTBDH) was overexpressed after combination treatment in infected macrophages (**Figure 2A**).

To reconfirm the biphasic characterization in the arachidonic acid (AA) pathway, the expression of enzymes involved in the AA pathway was investigated using real-time quantitative RT-PCR. The results revealed a significant enhancement of expression in cPLA2G4A, PTGES, and MPGES at 7.97, 8.61, and 7.74 fold compared to the combination therapy [**Figure 2B** (i-iv)]. Interestingly, combination therapy exhibited ALOX-5 and LTBDH expression at 13.11 and 8.11 fold significantly, compared to infected macrophages [**Figure 2B** (vi-vii)]. Therefore, our data support the notion that the expression of prostaglandin and leukotrienes was accomplished in opposite ways during infection and treatment. The enzymes responsible for prostaglandin synthesis were overexpressed in infection set to sustain parasite persistence while enzymes for leukotriene overexpressed in combination treatment to support parasite elimination from infected macrophages [**Figure 2B** (i-vii)].

### Effect of Combination Therapy of Eugenol Oleate and Amphotericin B in COX-2 Mediated Prostaglandin Synthesis and Leukotriene Release in Infected Macrophages

**Figure 3:**
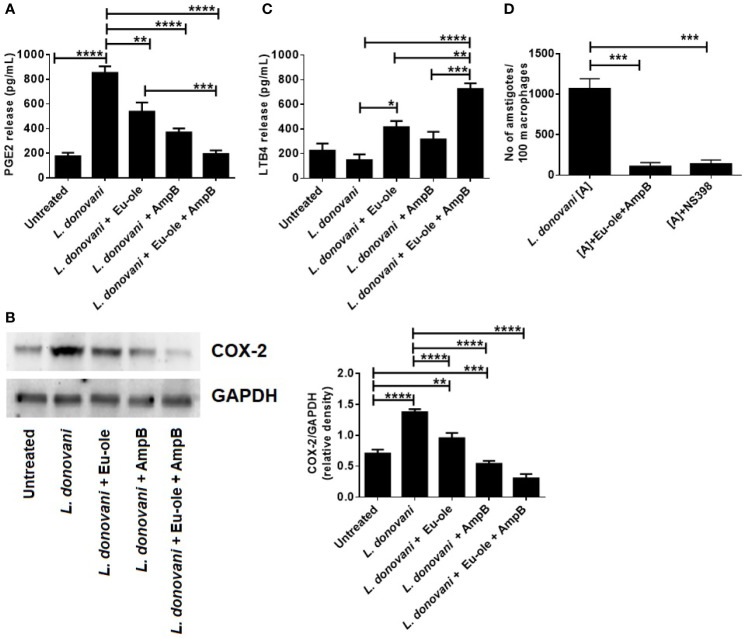
Effect of combination therapy in PGE2 release by COX-2 mediated pathway with induction of LTB4 release in infected macrophages. Cells were infected with *L. donovani* parasite at 1:10 ratio. After 24 hr of post infection treatment was given to macrophages. After 24 h of post treatment cell free supernatant was used to estimate PGE2 and LTB4 release by sandwich ELISA **(A, C)**. In a separate experiment after 24 hr of treatment at different concentration, the expression of COX-2 protein was assessed by immunoblotting assay, and the result of densitometry data is expressed as mean ± SD from triplicate experiments **(B)**. Cells were treated with combination of drugs or COX-2 inhibitor ((NS-398, 1µM). After 48 h of post treatment anti-amastigote study was performed by Giemsa staining method and represented as parasites per 100 macrophages **(D)**, *p *<* 0.05, **p *<* 0.01, ***p *<* 0.001, ****p *<* 0.0001, significant differences between the indicated groups.

It was previously reported that during Leishmania infection, COX-2 expression is induced for the induction of PGE2 release (Matte et al., 2001). As anticipated, it was found that the level of PGE2 was upregulated 4.38 fold during *L. donovani* infection, which was significantly higher compared to uninfected macrophages. During monotherapy treatment, PGE2 release was exhibited at 537.8 pg/ml and 369.7 pg/ml in eugenol oleate and amphotericin B respectively. Interestingly, combination-treated macrophages showed significant depletion in PGE2 release (194.6 pg/ml) (**Figure 3A**). Afterward, we analyzed the expression of COX-2, a regulatory enzyme for PGE2 release at the protein level. We observed that COX-2 expression was enhanced in obligatory *L. donovani* infected macrophages. Interestingly, it was noteworthy that the treatment of the synergic combination of eugenol oleate at 2.5 µM and 0.3125 µM of amphotericin B, COX-2 expression was significantly abrogated at protein levels in infected macrophages (**Figure 3B**). Decreased parasite load in the presence of COX-2 inhibitor (NS-398) as well as by combination therapy further confirmed that COX-2 inhibition helped in the intracellular parasite clearance (**Figure 3D**).

It was previously reported that leukotrienes, derived from the 5-lipo-oxygenase metabolism of the AA pathway, play an important role in controlling *Leishmania* infection (Chaves et al., 2016). To investigate the disease resolving the role of leukotrienes, we checked the level of LTB4 release in *L. donovani* infected macrophages. The treatment of combination therapy showed significant induction (725.2 pg/ml) in the level of LTB4 release in *L. donovani* infected macrophages *in vitro* (**Figure 3C**). Contrary to the findings of *in vitro* infections with *L. donovani* parasite, they showed a significant decrease in LTB4 production at 147.5 pg/ml. These *in vitro* findings indicate that the combination of eugenol oleate and amphotericin B could play a pivotal role in killing parasites by the release of leukotriene production by dampening the COX-2 mediated PGE2 release.

### Activation of MAPK in Immune Metabolic Alteration in *L. donovani* Infected BALB/c Derived Macrophages by NOS-2

**Figure 4:**
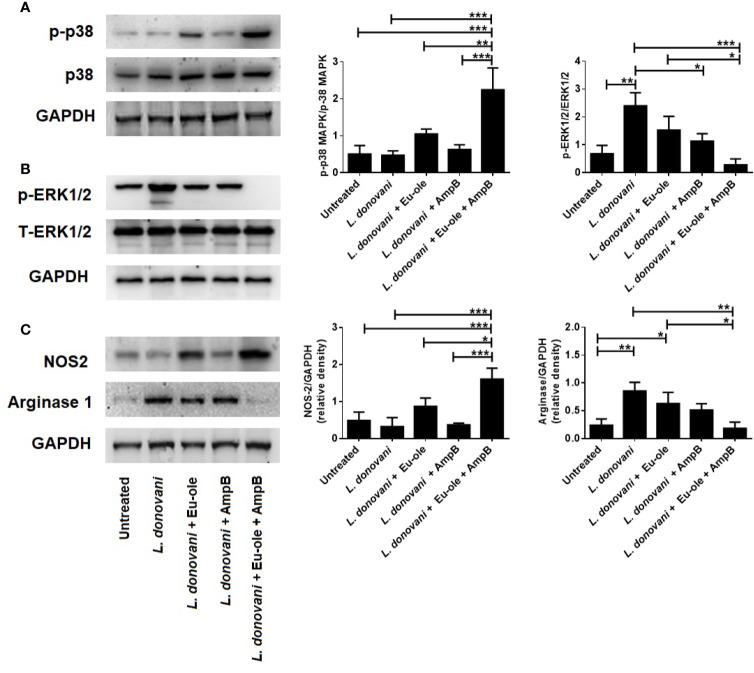
Effect of combination therapy in p38MAPK, ERK1/2 expression in *L. donovani* infected macrophages. BALB/c derived macrophages were infected *L. donovani* promastigotes followed by eugenol oleate (2.5 µM) and amphotericin B (0.3125 µM) alone or in combination. After 30 min of treatment, protein was extracted and subjected to western blotting to study the expression of p-p38MAPK, and p-ERK1/2 **(A, B)**. In a separate set of experiment, after 24 h of treatment, we performed the NOS-2 and arginase 1 expression by immunoblotting **(C)**. Densitometric analysis was expressed as mean ± SD from triplicate experiments. *p *<* 0.05, **p *<* 0.01, ***p *<* 0.001, significant differences between the indicated groups.

We know that the activation in p38MAPK phosphorylation attenuates *Leishmania donovani* infection in infected macrophages, as proven in several reports (Junghae and Raynes, 2002; Charan Raja et al., 2021). To explore the mechanistic pathway, we investigated whether the proposed combination of drugs could activate MAPK signaling in infected macrophages. After 30 min treatment, cells were collected in a chilled RIPA buffer for immunoblot analysis. **Figure 4A** indicates that combination therapy induced the phosphorylation of p-38MAPK in infected cells whereas infection by the *L. donovani* parasite is associated with the increased phosphorylation of ERK1/2 (**Figure 4B**). It was previously reported that the combination of eugenol oleate and amphotericin B increased NO generation at 30.91 µM by the expression of iNOS-2 in peritoneal exudate macrophages (Kar et al., 2021). The suppressive function of combination therapy is reciprocally associated with the expression of arginase-1 and iNOS following COX-2 mediated PGE2 production. As indicated in **Figure 4C**, NOS2 was highly expressed in combination-treated macrophages, whereas arginase-1 overexpressed only in infected macrophages.

### Anti-Amastigote Activity of Combination Therapy in Presence of Inhibitor

**Figure 5:**
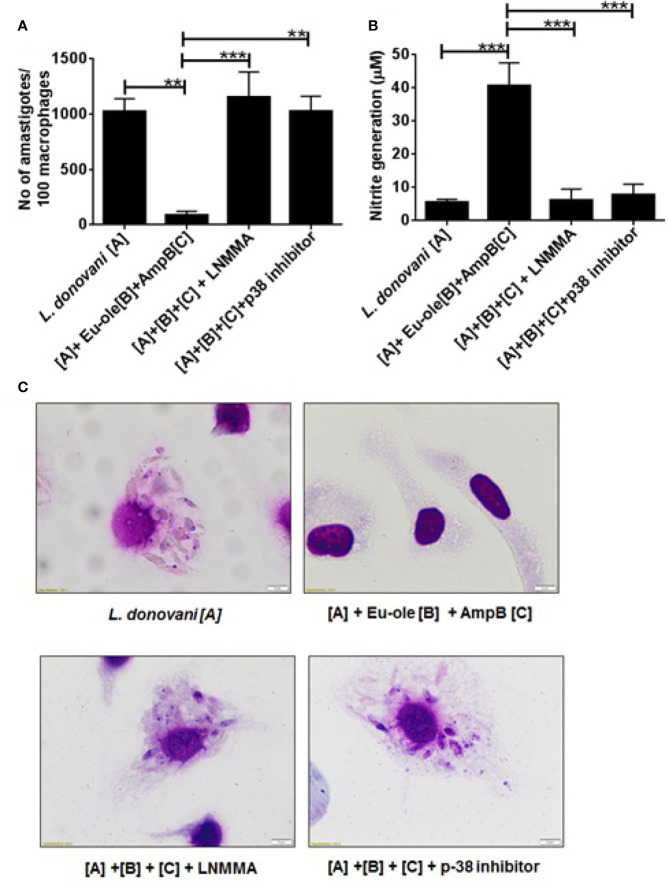
Effect of combination therapy in anti-leishmanial activity and NO generation in presence of inhibitors. Cells were pre-treated with p38 inhibitor (SB203580 at 5 μg/ml), iNOS inhibitor (*L*-NMMA at 0.4 mM) for 1 h followed by combination treatment. After 48 h of post treatment anti-amastigote study was performed by Giemsa staining method and NO generation was performed by Griess assay. **(A)** showed parasites per 100 macrophages, **(B)** showed NO generation in presence of inhibitor and **(C)** showed the representative macrophage images. **p *<* 0.01, ***p *<* 0.001, significant differences between the indicated groups.

To confirm the importance of NOS2 expression and p-38MAPK phosphorylation in combinatorial treatment with eugenol oleate and amphotericin B, we studied *in vitro* anti-amastigote activity and NO generation by Griess assay in peritoneal macrophages. The results confirmed that combination therapy indicated 91.42% of parasite-killing infected macrophages, while this parasite clearance was completely abrogated in the presence of SB203580 and LNMMA (**Figures 5A–C**). Simultaneously, the production of nitric oxide was also abolished in the presence of p38 and NO inhibitors in combinatorial treated cells (**Figure 5B**). In light of the above findings, we investigated that 2.5 µM of eugenol oleate could be involved in the alteration of immune metabolic reprogramming and parasite killing by p38MAPK induced NO generation in combination with 0.3125 µM of amphotericin B in *L. donovani* infected macrophages.

## Discussion

During evolutionary progress in new drug discovery and clinical manifestation towards *L. donovani* infection, several drugs like sodium stibogluconate, miltefosine, paromomycin, and amphotericin B are proposed for VL treatment (Hendrickx et al., 2017). Although evidence on the continued limitations with monotherapies reveals that the use of current therapies has to be reviewed. Combination therapy would be a better approach to overcome recent pitfalls in leishmaniasis treatment. Several synthetic compounds in combination with the standard drugs could be useful for long-term efficacy in treatment (Zulfiqar et al., 2017). Likewise, eugenol oleate, a derivative of eugenol, could be a better option for its immunomodulatory effect (Charan Raja et al., 2017). Recently, we observed that eugenol oleate showed synergistic interaction in combination with amphotericin B against intracellular *L. donovani* amastigotes (x∑FIC = 0.456). In this study, we selected the concentration of eugenol oleate at 2.5 µM in combination with 0.3125 µM of amphotericin B, which indicates 94.93% parasite killing against *L. donovani* intracellular amastigotes. Furthermore, we evaluated that association of these two compounds had better efficacy against experimental VL *in vitro* and *in vivo* (Kar et al., 2021).

The present study explored the mechanism of combination therapy in the modulation of host immune metabolism during *L. donovani* infection and the further consequences on the host-protective immune response. The present study examined this using combination therapy with 2.5 µM of eugenol oleate and 0.3125 µM of amphotericin B induced glycolytic enzyme in infected macrophages (**Figures 1A–B**). Glycolysis is an essential metabolic event that provides energy to survive and converts glucose into pyruvate (Gonçalves et al., 2019). During glycolysis, hexokinase coverts glucose into glucose-6-phosphate by several intermediates (Tan and Miyamoto, 2015). In macrophages, the glucose was uptaken by its glucose transporter GLUT-1, which provides energy to facilitate glucose (Macintyre et al., 2014). Qualitative RT-PCR data indicated that combination therapy exhibited increased expression of GLUT-1 and hexokinase 1, hexokinase 2, and hexokinase-3 significantly, compared to the infected macrophages (**Figure 1A**). Hexokinase catalyzed glucose phosphorylation into glucose-6-phosphate during glycolysis metabolism (Marko et al., 2010). Moreover, phosphorylated hexokinase pulled out glucose from equilibrium to trap inside the cell. For this reason, hexokinase is an important regulator for glycolysis. Additionally, glucose uptake was dependent upon the upregulation of GLUT-1 expression (Wang et al., 2017). In addition, the expression of PFKB3 and PFKM transcript was induced during combination therapy (**Figure 1B** (v-vi)). Indeed, these two enzymes PFKB3 and PFKM regulated macrophage metabolism and polarization. Despite this, it was detected that upon challenge with *L. infantum* parasite, the level of glycolytic enzymes was switched to the early stage of infection but not after the late phase of infection (Moreira et al., 2015). Not only that, *L. amazonensis* used the host metabolism for their survival and eventually converted into M2 polarized macrophages (Ty et al., 2019). In our present study, we investigated that the given combination of two compounds, eugenol oleate, and amphotericin B could provide energy to the host cellular metabolism to shift from VL progressing M2 to VL, resolving M1 macrophages. Extensive studies on glycolysis for cellular metabolism remain elusive and further studies are required to reach clear conclusions.

To understand the immune metabolic reprogramming during *Leishmania* infection, we also evaluated the release of arachidonic acid metabolites including leukotrienes and prostaglandins in infected peritoneal macrophages. In subversion by *L. donovani* infection, parasites utilize anti-inflammatory cytokines (TGF-β, IL-10) and arachidonic acid metabolites (predominantly prostaglandins) to survive inside macrophages (Bodhale et al., 2020). Subsequently, we investigated that the release of prostaglandin was regulated by the cyclooxygenase (COX-2) pathway during *L. donovani* infection (**Figures 3A–B**). Interestingly, upon treatment of combination therapy, the level of prostaglandin was inhibited, whereas the production of leukotrienes was restored to impair the intracellular parasite function. While LTBDH exhibited moderately higher expression in combination therapy in treated macrophages (**Figure 2A**). As expected, RT-PCR data also suggested that the enzymes involved in the prostaglandin synthesis pathway (PTGER4, MPGES, and PTGES) were highly expressed in infected macrophages [**Figure 2B** (iii-v)].

Several studies have reported that the prostaglandin (PGE2) production was elevated after 24 hr of post-infection by *Leishmania* parasites (Saha et al., 2014). It has been rpeviosuly demonstrated that the combination of 2.5 µM of eugenol oleate with 0.3125 µM of amphotericin B was capable of shifting from the Th-2 response to VL, resolving Th-1 response against *L. donovani*. Additionally, macrophage activation induced by the mentioned combination was accompanied by the production of pro-inflammatory cytokines such as IL-12, TNF-α, and IFN-γ and anti-inflammatory agents IL-10 and TGF-β (Kar A et al., 2021). Our findings indicated that COX-2 generated PGE2 production was augmented in *L. donovani* infected macrophages. Several studies have observed that IL-10 contributed to PGE2 signaling through the upregulation of E-type prostaglandin receptors (EP4) *via* STAT3 (Samiea et al., 2020). Eventually, the release of PGE2 production was restricted by the treatment of synergistically combined molecule eugenol oleate and amphotericin B (**Figure 3A**). In BALB/c derived macrophages, COX-2 mediated PGE2 release was shown to release anti-inflammatory cytokines IL-10, which downregulated proinflammatory cytokine production and macrophage activation. Interestingly, the recruitment of combination therapy in infected macrophages resulted in induced leukotriene production along with the host-protective immune response (**Figure 3C**). Thus, it could be concluded that this combination might involve in leukotriene production with the inhibition of PGE2 release.

The present study examined the alteration in the expression of glycolytic enzymes and biphasic nature in the arachidonic acid metabolism pathway. Apart from these findings in the immune metabolic status of *L. donovani* infected macrophages and the impact of combination therapy with eugenol oleate and amphotericin B, we investigated the involvement of MAPK signaling after combination therapy in infected peritoneal macrophages. We revealed that combination therapy mediated the implications of the MAPK signaling pathway by demonstrating that eugenol oleate in combination with amphotericin B induced the phosphorylation of p38MAPK, and abolished the phosphorylation of ERK1/2 (**Figures 4A, B**). In another recent study, we discussed the role of combination therapy as an anti-leishmanial agent against the *L. donovani* parasite, both *in vivo* and *in vitro* through NO generation and upregulation in pro-inflammatory cytokines (Kar et al., 2021). It is now well established that the drug-mediated phosphorylation of p38 could promote NO generation during *L. donovani* infection (Wadhone et al., 2009; Charan Raja et al., 2021). Hence, the combination of eugenol oleate and amphotericin B modulated phosphorylation of p38MAPK might be involved in iNOS-2 mediated NO generation in infected macrophages.

In the immunoblotting study, we also found that the combinatorial treatment of *L. donovani*-infected macrophages induced the iNOS-2 expression and reduced the expression of arginase-1 (**Figure 4C**). We further investigated that there were no alterations in amastigote numbers per macrophage and NO production with pre-treated p38inhibitor (SB352080) and NO inhibitor (LNMMA) in infected macrophages even after combination therapy with eugenol oleate and amphotericin B (**Figures 5A–C**). Collectively, the *in vitro* data from the present study elucidated that the combination of eugenol oleate and amphotericin B increased iNOS-2 expression primitively dependent on p38 MAPK and iNOS-2 dependent NO generation played a master role in the restoration of immune functions against *L. donovani* infection.

**Figure 6:**
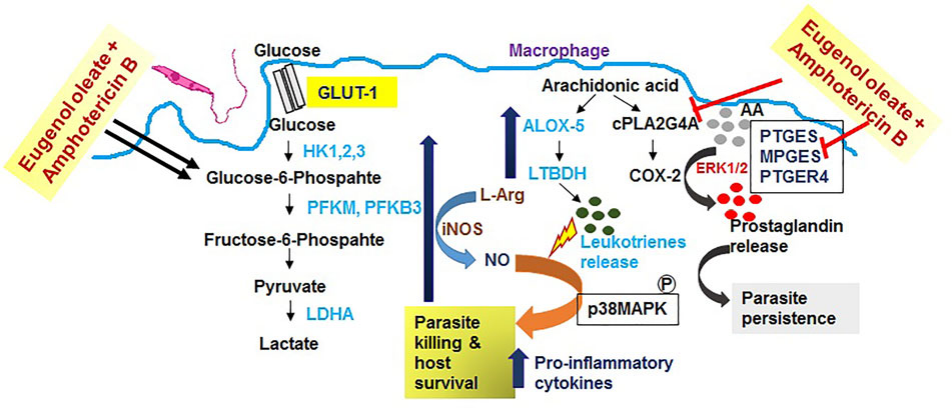
Schematic diagram of the possible mechanism of combination therapy with eugenol oleate and amphotericin B.

Corroborating our present findings in this *in vitro* study, we observed a possible adaptation in immune metabolic flux during the combination therapy of eugenol oleate and amphotericin B in infected BALB/c derived macrophages (**Figure 6**). Further studies should be undertaken to enable a better understanding of this host immune status, meaning that combination therapy with amphotericin B with eugenol oleate becomes an acceptable approach for VL treatment in the near future.

## Data Availability Statement

The raw data supporting the conclusions of this article will be made available by the authors, without undue reservation.

## Ethics Statement

The animal study was reviewed and approved by CPCSEA Reg. No. 817/PO/ReRcBiBt/S/04/CPCSEA; Dated 13.12.2018 and was approved by the ethical committee of SASTRA Deemed to Be University (612/SASTRA/IAEC/RPP; dated 10.08.2019).

## Author Contributions

AK: Conceptualization, Methodology, Validation, Investigation, Formal Analysis, Data Curation, Writing - Original Draft, Writing - Review and Editing. AJ: Methodology, Validation, Formal Analysis. AK: Methodology, Validation. SK: Conceptualization, Methodology, Formal Analysis, Resources, Writing - Original Draft, Writing - Review and Editing, Visualization, Supervision, Project Administration, Funding Acquisition. All authors contributed to the article and approved the submitted version.

## Funding

AK is thankful to FNDR, Bangalore India (G170021) for the fellowship.

## Conflict of Interest

The authors declare that the research was conducted in the absence of any commercial or financial relationships that could be construed as a potential conflict of interest.

## Publisher’s Note

All claims expressed in this article are solely those of the authors and do not necessarily represent those of their affiliated organizations, or those of the publisher, the editors and the reviewers. Any product that may be evaluated in this article, or claim that may be made by its manufacturer, is not guaranteed or endorsed by the publisher.
