# Supplementary material for: Dynamicity in Host Metabolic Adaptation Is Influenced by the Synergistic Effect of Eugenol Oleate and Amphotericin B During Leishmania donovani Infection In Vitro
Source: Front Cell Infect Microbiol. 2021 Aug 3;11:709316. doi: 10.3389/fcimb.2021.709316 (PMC8369346; doi:10.3389/fcimb.2021.709316)

Supplementary data/information

Dynamicity in host metabolic adaptation is influenced by the synergistic effect of eugenol oleate and amphotericin B during *Leishmania donovani* infection *in vitro*

Amrita Kar,[1] Adithyan Jayaraman,[1] Avanthika Kumar,[1] Santanu Kar Mahapatra[1,2]*

[1]*Department of Biotechnology, School of Chemical and Biotechnology, SASTRA Deemed to be University, Thanjavur-613401, India.*

[2]*Department of Paramedical and Allied Health Sciences, Midnapore City College, Midnapore-721129, West Bengal, India.*

Table 1:

Primer sequences:

Gene	Primer sequences
GLUT1	Forward 5′ GCT GTG CTT ATG GGC TTC TC 3′;<br>Reverse 5′ AGA GGC CAC AAG TCT GCA TT 3′;
HK-1	Forward 5′ GCT CAG AAA AGG GGG ATT TC 3′;<br>Reverse 5′ TCA AAA AGC TGG CTT CCA CT 3′;
HK-2	Forward 5′ GGG TAG CCA CGG AGT ACA AA 3′;<br>Reverse 5′ TGG ATT GAA AGC CAA CTT CC 3′;
HK-3	Forward 5′ AGA CAT TGC AAG TGG CTG TG 3′;<br>Reverse 5′ ACT GTC AGC TGC TCC AAG GT 3′;
PFKM	Forward 5′ CTG CAG GTG AAG GAG AAA GG 3′;<br>Reverse 5′ AGA TGC AAA CAC CAT GTC CA 3′;
PFKB-3	Forward 5′ GGC TGT TCT ACG CTG CCT AC 3′;<br>Reverse 5′ GGG TTA GGT CCC TTC TTT GC 3′;

LDHA	Forward 5′ CCG TTA CCT GAT GGG AGA GA 3′;<br>Reverse 5′ GTA GGC ACT GTC CAC CAC CT 3′;
cPLA2G4A	Forward 5′ ACC CCT TGC ATT TCT TGA TG 3′;<br>Reverse 5′ ACC CAA CTT GCT TGG TTG TC 3′;
COX-2	Forward 5′ GGA GAG ACT ATC AAG ATA GTG ATC 3′;<br>Reverse 5′ ATG GTC AGT AGA CTT TTA CAG CTC 3′
PTGES	Forward 5′ CCT TGA GCT GAC AGC CTA CC 3′;<br>Reverse 5′ GCC ACA TGG AAG ACA GGA TT 3′ ;
MPGES	Forward 5′ CCT AGG CTT CAG CCT CAC AC 3′;<br>Reverse 5′ CAG CCT AAT GTT CAG CGA CA 3′;
PTGER4	Forward 5′ GAT GGC TGA GGT TGG AGG TA 3′;<br>Reverse 5′ CCC CGA AGA TGA ACA TCA CT 3′;
ALOX-5	Forward 5′ CTA CGA TGT CAC CGT GGA TG 3′;<br>Reverse 5′ GTG CTG CTT GAG GAT GTG AA 3′;
LTBDH	Forward 5′ CAT GAG AGT TGC AGC CAA AA 3′;<br>Reverse 5′ GGC AGC TTT GTC AGT CCA TT 3′;
GAPDH	Forward 5′ GAG CCA AAC GGG TCA TCA TC 3′;<br>Reverse 5′ CCT GCT TCA CCA CCT TCT TG- 3′;

Western Blotting images:

Figure 3B: COX2

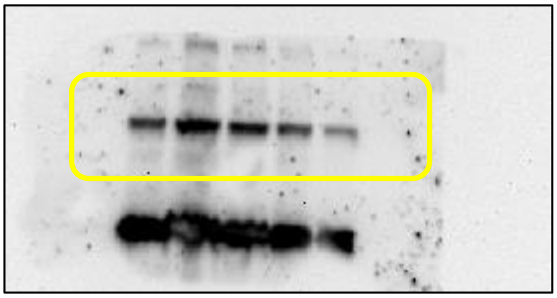

Figure 3B: GAPDH

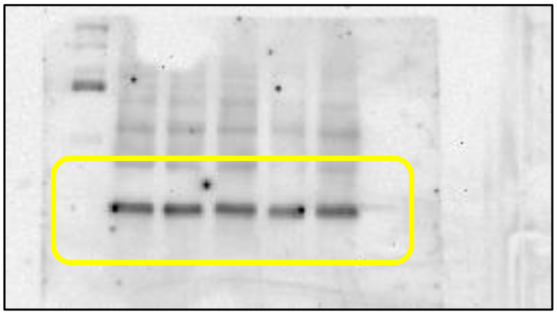

Figure 4A: p-p38

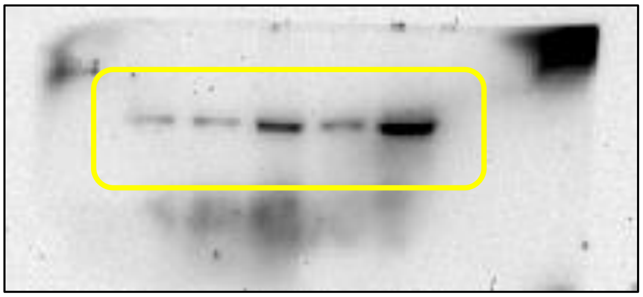

T-p38:

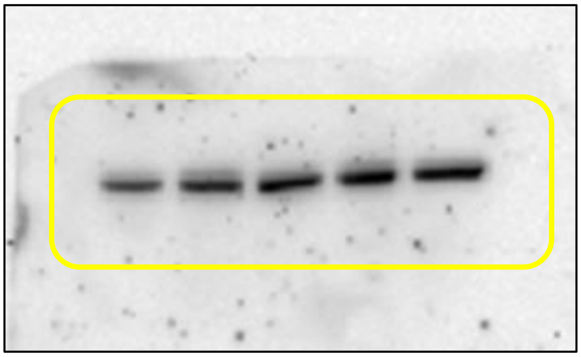

GAPDH:

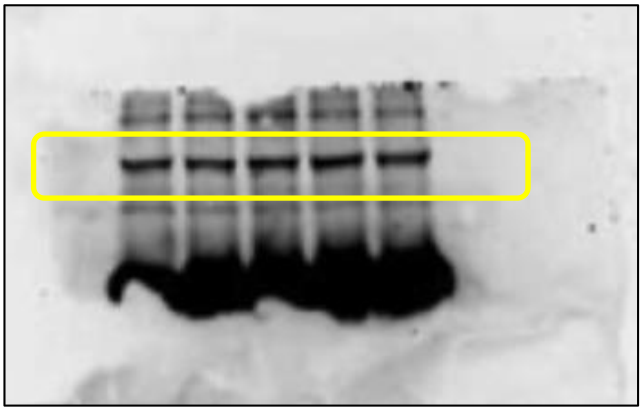

Figure 4B:

p-ERK1/2:

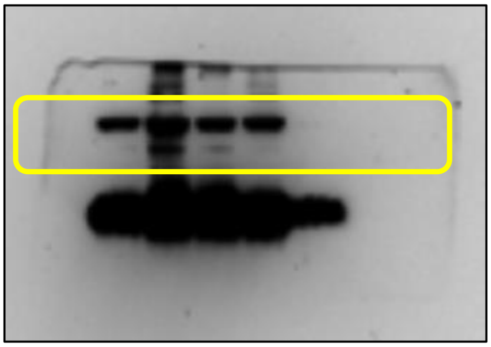

T-ERK

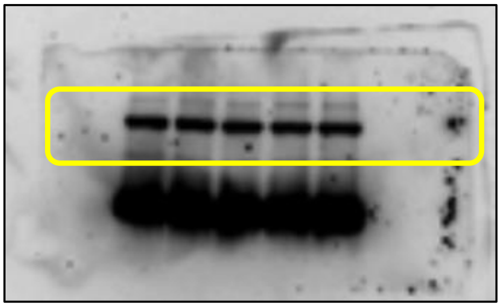

GAPDH:

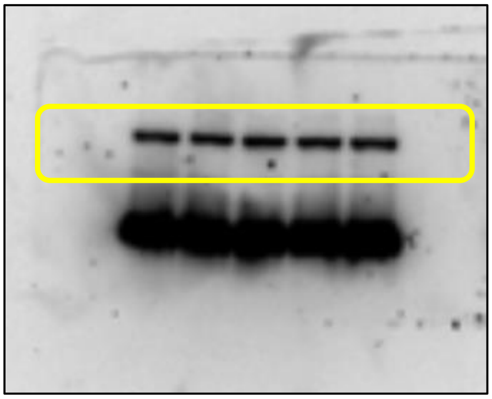

Fig. 4C: NOS2

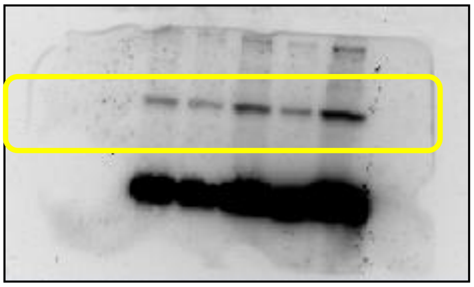

Arginase

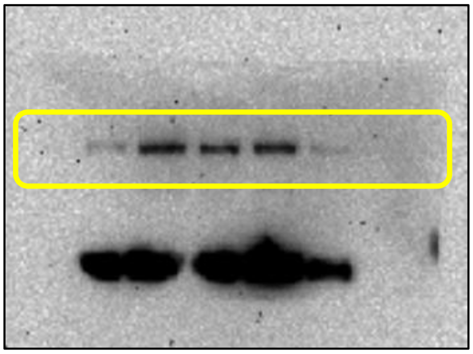

GAPDH

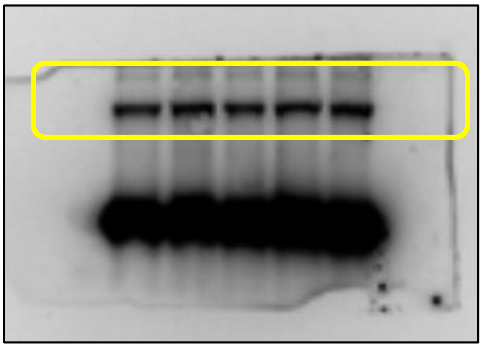

Supplement: Supplementary file 1 [file DataSheet_1.pdf]
